# Effects of LMW-GS Allelic Variations at the *Glu-A3* Locus on Fresh Wet Noodle and Frozen Cooked Noodle Quality

**DOI:** 10.3390/foods14091546

**Published:** 2025-04-28

**Authors:** Xiaohong Chen, Hongwei Zhou, Yufei Zou, Jinfu Ban, Huizhi Zhang, Xiaoke Zhang, Boli Guo, Yingquan Zhang

**Affiliations:** 1 Institute of Food Science and Technology, Chinese Academy of Agricultural Sciences/Comprehensive Utilization Laboratory of Cereal and Oil Processing, Ministry of Agriculture and Rural, Beijing 100193, China; chenxiaohong0914@163.com (X.C.); zhouhw12356@163.com (H.Z.); yfzou1218@163.com (Y.Z.); zhz18638707897@163.com (H.Z.); 2 College of Agronomy, Northwest A & F University, Yangling 712100, China; zhangxiaoke66@nwafu.edu.cn; 3 Shijiazhuang Academy of Agricultural and Forestry Sciences, Center of Wheat Research, Shijiazhuang 050041, China; sjznkybjf@163.com; 4 Institute of Western Agriculture, The Chinese Academy of Agricultural Sciences, Changji 831100, China

**Keywords:** wheat, LMW-GS, *Glu*-*A3*, near-isogenic lines, noodle quality

## Abstract

Low molecular weight glutenin subunits (LMW-GSs) in wheat are critical functional proteins that regulate the processing quality of flour-based products. This study utilized two sets of near-isogenic lines (NILs) derived from the wheat cultivars Zhoumai 22 and Zhoumai 23 to investigate the effects of allelic variations at the *Glu-A3* locus—specifically *Glu*-*A3a*, *Glu*-*A3b*, *Glu*-*A3c*, *Glu*-*A3d*, *Glu*-*A3e*, *Glu*-*A3f*, and *Glu*-*A3g*—on protein content, gluten properties, dough farinograph properties, cooking properties of fresh wet noodles (FWNs), and textural properties of FWNs and frozen cooked noodles (FZNs). The results demonstrated that *Glu*-*A3f* exhibited superior grain protein content. *Glu-A3e* negatively impacted the gluten index, and *Glu*-*A3g* showed favorable dry gluten content. *Glu*-*A3b* displayed enhanced dough mixing tolerance. Importantly, *Glu*-*A3b* was associated with improved hardness in FWNs, while *Glu*-*A3g* contributed to higher hardness and chewiness in FZNs. These findings provide critical insights for breeding elite wheat cultivars tailored for noodle production and optimizing specialty flour development.

## 1. Introduction

Common wheat (*Triticum aestivum* L.) is one of the most vital global crops, with approximately 12% of annual wheat production dedicated to manufacturing diverse noodle products [1,2]. Fresh wet noodles (FWNs) are increasingly favored by consumers because of their rich nutrition, natural flavor, and good taste [3]. Frozen cooked noodles (FZNs) are a non-fried, low-temperature stored instant noodle product with the characteristics of fast thawing, smooth taste, and excellent flavor. They have huge market potential and are gradually becoming the representative of a new generation of noodles [4]. Wheat flour is uniquely suited for creating noodles with distinct quality and flavor due to its viscoelastic gluten network. Gluten properties are primarily determined by the composition and quantity of glutenin and gliadin [5]. Glutenin is categorized into high molecular weight glutenin subunits (HMW-GSs) and low molecular weight glutenin subunits (LMW-GSs). While allelic variations in HMW-GSs are relatively easy to isolate and characterize, extensive research has focused on their contributions to dough, bread, and noodle quality [2,6,7]. LMW-GSs account for approximately one-third of wheat storage proteins [8]. Most genes encoding LMW-GSs are located on the short arms of chromosomes 1A, 1B, and 1D within the first homologous group, specifically at the *Glu*-*A3*, *Glu*-*B3*, and *Glu*-*D3* loci. Each locus harbors multiple LMW-GS genes, forming a complex multigene family [9,10,11], which complicates the isolation and identification of LMW-GS allelic variants. Fortunately, Wang et al. [12] developed seven dominant allele-specific sequence-tagged site (STS) markers to efficiently distinguish *Glu*-*A3* allelic variants (*Glu*-*A3a*, *Glu*-*A3b*, *Glu-A3c*, *Glu-A3d*, *Glu-A3e*, *Glu-A3f*, and *Glu-A3g*), providing a robust foundation for studying the influence of allelic variation on wheat quality traits.

At present, the main results of the effect pattern of the *Glu-A3* locus on wheat quality characteristics are as follows. He et al. [13] found that the effect size of LMW-GS allelic variants at the *Glu-A3* locus on dough stability time was *Glu-A3d* > *Glu-A3c*/*Glu-A3a* using 76 main and high-generation varieties as materials. Zhang et al. [14], using Aroona near-isogenic lines (NILs), found that the ranking of *Glu*-*A3* LMW-GS allelic variants’ effects on dough stability time was *Glu*-*A3b*/*Glu*-*A3d*/*Glu*-*A3f*/*Glu*-*A3c* > *Glu*-*A3e*. Guzmán et al. [1], analyzing 4623 grain samples from 2550 genotypes, demonstrated that the ranking for optimal dough mixing time was *Glu*-*A3b*/*Glu*-*A3d*/*Glu*-*A3f* ≥ *Glu*-*A3g* ≥ *Glu*-*A3c*/*Glu*-*A3e*. He et al. [13] reported that *Glu*-*A3* LMW-GS alleles influenced dry white noodle scores as *Glu*-*A3d* ≥ *Glu*-*A3c* ≥ *Glu*-*A3a*, while Jin et al. [15], employing Aroona NILs, ranked their effects on fresh wet noodles’ color parameter b* as *Glu*-*A3e* ≥ *Glu*-*A3c* ≥ *Glu*-*A3d*/*Glu*-*A3f* ≥ *Glu*-*A3b*. Zhou et al. [16], using Xiaoyan 22 NILs, observed that the impact of *Glu*-*A3* alleles on FWN quality followed *Glu*-*A3c*/*Glu*-*A3e* > *Glu*-*A3a*/*Glu*-*A3b*. Notably, previous studies of the contribution of the same allelic variations to dough quality were contradictory, e.g., on the dough quality of *Glu*-*A3d* and *Glu*-*A3c*, and the effects of LMW-GS allelic variations on the qualities of FWNs and frozen cooked noodles (FZNs) have been less reported.

NILs refer to a group of lines that have the same or similar genetic background but differ in a target gene. Therefore, NILs are ideal for comparative studies of the contribution size of allelic variation [17]. China is the largest producer and consumer of wheat in the world, and the Yellow and Huai Valley has the highest sown area and yield of wheat in China. The wheat varieties Zhoumai 22 and Zhoumai 23 are currently widely promoted in the Yellow and Huai Valley wheat areas. With the background of the current main popularized varieties, we investigated the influence of LMW-GS allelic variants on wheat quality, which is of more practical significance for the improvement of wheat quality [18]. In this study, two sets of NIL materials constructed from the genetic background of the wheat varieties Zhoumai 22 and Zhoumai 23 were selected to analyze the effects of the LMW-GS allelic variants *Glu-A3a*, *Glu-A3b*, *Glu-A3c*, *Glu-A3d*, *Glu-A3e*, *Glu-A3f*, and *Glu-A3g* at the *Glu-A3* locus on the wheat’s protein content, gluten characteristics, dough farinograph traits, cooking properties of FWNs, and textural properties of FWNs and FZNs, to provide a basis and reference for the selection and breeding of high-quality wheat varieties specializing in noodles and optimizing specialty flour development.

## 2. Materials and Methods

### 2.1. Experimental Materials

In this study, 2 sets of NIL materials constructed from the genetic background of wheat varieties Zhoumai 22 and Zhoumai 23 were selected, whose *Glu-A3* locus’ LMW-GSs carry *Glu-A3a*, *Glu-A3b*, *Glu-A3c*, *Glu-A3d*, *Glu-A3e*, *Glu-A3f*, and *Glu*-*A3g* allelic variants. For simplicity, the 7 NILs in the same background were abbreviated as A3a, A3b, A3c, A3d, A3e, A3f, and A3g. Zhoumai 22 (*Glu-A3d* and *Glu-B3j*) was used as the recurrent parent, and the Chinese Spring (*Glu-A3a* and *Glu-B3a*), Shandong 413863 (*Glu-A3b* and *Glu-B3j*), Yuwai 69 (*Glu-A3c* and *Glu-B3d*), CA9641 (*Glu-A3d* and *Glu-B3h*), Jinnong 207 (*Glu-A3e* and *Glu-B3j*), Yuandong 6 (*Glu-A3f* and *Glu-B3j*), and Gaocheng 8901 (*Glu-A3g* and *Glu-B3i*) were used as non-recurrent parents; when Zhoumai 23 (*Glu*-*A3d* and *Glu*-*B3d*) was the recurrent parent, China Spring (*Glu*-*A3a* and *Glu*-*B3a*), Shandong 413863 (*Glu*-*A3b* and *Glu*-*B3j*), CA9719 (*Glu*-*A3c* and *Glu*-*B3h*), Nongda 116 (*Glu*-*A3d* and *Glu*-*B3d*), Jinong 207 (*Glu*-*A3e* and *Glu*-*B3j*), Nongda 3213 (*Glu*-*A3f* and *Glu*-*B3j*) and Gaocheng 8901 (*Glu*-*A3g* and *Glu*-*B3i*) were used as non-recurrent parents (Table S1). STS molecular markers were utilized to select the target subunits during the backcross breeding process. Zhoumai 22’s parents are Wenmai 6, Zhoumai 13, and Zhoumai 12, with genetic contribution rates of 37.25%, 36.14%, and 26.15%, respectively. Zhoumai 23’s parents are Zhoumai 13 and Xinmai 9, with genetic contribution rates of 63.04% and 36.96%, respectively. Although Zhoumai 22 and Zhoumai 23 share a common parent, Zhoumai 13, there are also significant genetic background differences between the 2 varieties.

### 2.2. Field Experiment Design

The field experiment was conducted in October 2022 at Yangling, Shaanxi Province, China (108°10′ E, 34°30′ N), in a randomized complete block design (RCBD) with 2 replications. The area of each replication was 8 square meters. The experiment was sown by hand at a spacing of 0.25 m between rows and 0.022 m between plants. According to the local field fertilization level, 750 kg/ha of nitrogen, phosphorus, and potassium ternary compound fertilizer (total nutrient content ≥ 42%, containing 17%–20% N, 18%–20% P, and ≥5% K); 2400 kg/ha of commercial granular organic fertilizer (N + P + K ≥ 5%, and organic matter content > 45%); and 60 kg/ha of insecticide (effective content of 5%, of which 2% was chlorpyrifos and 3% was phoxim). The experimental plots were winter-irrigated in January 2023. The plots were harvested using a small plot harvester in June 2023. Before milling, the seeds were dried and placed in a mesh bag, sealed with plastic sheets, and stored at room temperature for 2 months.

### 2.3. Milling

Seeds from 2 replicates were uniformly mixed for milling. The grains were soaked in a sealed container for 24 h to adjust the moisture content to 15.5%. Milling was performed using a Bühler MLU-202 laboratory mill (Bühler, Uzwil, Switzerland). After milling, a 50-mesh standard test sieve was used to screen the wheat flour. Under the Zhoumai 22 genetic background, the flour yields of NILs A3a, A3b, A3c, A3d, A3e, A3f, and A3g were 71.10%, 68.94%, 69.32%, 66.31%, 68.60%, 70.14%, and 67.63%, respectively. In the Zhoumai 23 background, the flour yields of NILs A3a, A3b, A3c, A3d, A3e, A3f, and A3g were 63.74%, 63.28%, 62.71%, 64.71%, 61.45%, 64.63%, and 66.37%, respectively.

### 2.4. Experimental Methods

#### 2.4.1. HMW-GSs and Gliadins

HMW-GSs and gliadin were extracted and isolated following the methodology described by Zhou et al. [16]. HMW-GS composition was analyzed using Sodium Dodecyl Sulfate Polyacrylamide Gel Electrophoresis (SDS-PAGE). The stacking and separating gels were prepared at concentrations of 4% and 8.7%, respectively. Electrophoresis was conducted at 11 mA per gel for 11 h. post-electrophoresis processing included fixation with a fixing solution, staining with Coomassie Brilliant Blue, and destaining with tap water to visualize HMW-GS protein bands. Gliadin composition was determined by acid-polyacrylamide gel electrophoresis (A-PAGE). Both stacking and separating gels were prepared at 5% concentration. Electrophoresis was performed at 20 mA per gel for 8 h, followed by staining and tap water destaining to reveal gliadin bands. The HMW-GS and gliadin profiles of NILs were compared against those of the recurrent parent, which served as the control.

#### 2.4.2. LMW-GSs

Genomic DNA was extracted from wheat leaves of 14 lines using the cetyltrimethylammonium bromide (CTAB) method. Target sequences were amplified using sequence-tagged site (STS) markers developed by Francis et al. [19] and Wang et al. [12,20] (Table 1), with genomic DNA as the template. Allelic variations of LMW-GSs at the *Glu*-*A3* locus in different NILs were identified based on the electrophoretic fragment sizes of the amplified products.

#### 2.4.3. Protein Content

The grain protein content (GPC) and flour protein content (FPC) were determined using a DA9500 near-infrared analyzer (Perten Instruments, Hägersten, Sweden) following the protocol described by Guo et al. [21].

#### 2.4.4. Gluten Characteristics

The wet gluten content (WGC), dry gluten content (DGC), and gluten index (GI) were measured according to the AACC International Method 38-12.00 (2000) [22], using a Gluten Quantity and Quality Index System (Perten Instruments, Hägersten, Sweden).

#### 2.4.5. Farinograph Properties

Farinograph parameters were evaluated according to the AACC International Method 54-21.00 (2000) [23], using a farinograph (Anton Paar Brabender GmbH & Co. KG, Duisburg, Germany) equipped with a 300g mixer bowl. The measured parameters included water absorption (WA), development time (DT), stability time (ST), degree of softening (DS), and farinograph quality number (FQN).

#### 2.4.6. Fresh Wet Noodles (FWNs) and Frozen Cooked Noodles (FZNs) Preparation

A total of 200 g of wheat flour, 2 g of salt, and an appropriate amount of water were mixed for 4 min to form dough crumbs in a needle-type dough mixer (Dongfu Jiuheng Instrument Technology Co., Ltd., Beijing, China). The water addition was calculated using the following formula: 200 × (0.35 − flour moisture content)/(1 − 0.35). The dough crumbs were sheeted at a gap of 2.5 mm, and then the dough was folded in half and continuously sheeted at the same gap of 2.5 mm 2 times in a noodle machine (Beijing Dongfu Jiuheng Instrument Technology Co., Ltd., China). The dough sheet was sealed in a self-sealing bag and rested at 25 °C for 30 min. Thereafter, the dough sheet was sheeted sequentially through gaps of 2 mm, 1.5 mm, 1 mm, 0.7 mm, and 0.5 mm to obtain a 1 mm thick sheet, and then it was cut into 1 mm wide FWNs. The noodles were boiled until 30 s before the optimal cooking time, cooled in cold water for 30 s, and immediately frozen in a −40 °C freezer for 1 h, then transferred to a −18 °C freezer and stored for 7 days to obtain FZNs. Each sample underwent 2 physical repetitions.

#### 2.4.7. Cooking Properties of Noodles

Noodle cooking properties, including the optimal cooking time (OCT), water absorption ratio (WAR), and cooking loss ratio (CLR), were determined according to the method described by Zhou et al. [15]. A total of 10 fresh noodle strands were cooked in 500 mL of boiling water. The OCT was recorded as the time when the white core of the noodles disappeared, monitored at 15s intervals. For WAR determination, 10 g of noodles (M_0_) were cooked in 500 mL of distilled water (M_1_) heated to boiling and maintained at a gentle boil using an induction cooker. After cooking to OCT, the noodles were rinsed under cold running water for 10 s, surface moisture was removed by blotting with absorbent paper, and the mass (M_2_) was weighed to the nearest 0.01 g. Then, the noodles’ soup was boiled until the liquid evaporated and it was dried to a constant weight in a drying oven at 130 °C. The final weight of the dried basin was recorded as M_3_. The cooking characteristics were repeated twice. WAR and CLR were calculated using Equation (1) and Equation (2), respectively:

Water absorption ratio (%) = (M_2_ − M_0_)/M_0_ × 100% (1)

Cooking loss ratio (%) = (M_3_ − M_1_)/(M_0_×(1 − ω)) × 100% (2)

where “ω” is the water content of FWNs.

#### 2.4.8. Texture Characteristics of Cooked Noodles

The texture properties of FWNs and FZNs were measured using a TA.XT Plus texture analyzer (Stable Micro Systems, Godalming, UK). The FWNs were cooked to their optimal cooking time, and the FZNs were directly boiled in boiling water for 1 min without thawing, followed by cooling in cold water for 15 s. Five noodle strands were aligned parallel on the testing platform for Texture Profile Analysis (TPA). The hardness, adhesiveness, springiness, cohesiveness, resilience, and chewiness of TPA parameters were recorded. The parameters were set as follows: TPA mode, ALKB-F probe, pre-test speed/test speed/post-test speed of 0.8 mm/s, 0.8 mm/s, and 2 mm/s, respectively, compression ratio of 70%, 5 s interval between 2 compressions, trigger force of 10 g, and data acquisition rate of 200 pps. Each repetition was measured five times.

### 2.5. Data Analysis

Experimental data were analyzed using SPSS Statistics 24.0 (IBM Corp., Armonk, NY, USA) with a one-way analysis of variance (ANOVA) followed by Duncan’s multiple range test for post hoc comparisons. Data normality was verified via the Shapiro–Wilk test (α = 0.05). Normally distributed data are expressed as mean ± standard deviation (mean ± SD), while non-normally distributed values are presented numerically. Visualizations were generated using OriginPro 2022 (OriginLab Corp., Northampton, MA, USA). Statistical significance was defined at *p* < 0.05.

## 3. Results

### 3.1. Identification of Gluten Protein Composition for NILs

**Figure 1:**
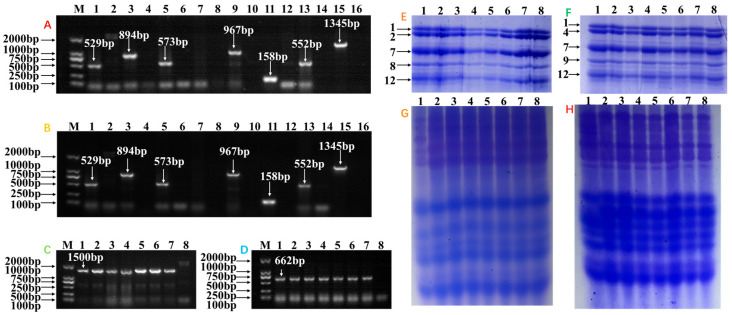
Identification of gluten protein compositions (**A**,**B**): LMW-GS composition of the *Glu-A3* locus. Lane M, marker; Lane 1, *Glu*-*A3a*; Lane 3, *Glu*-*A3b*; Lanes 5 and 7, *Glu*-*A3c*; Lane 9, *Glu*-*A3d*; Lane 11, *Glu*-*A3e*; Lane 13, *Glu*-*A3f*; Lane 15, *Glu*-*A3g*; Lanes 2, 4, 6, 8, 10, 12, 14, and 16, H_2_O. (**C**,**D**): LMW-GS composition at the *Glu*-*B3* locus. Lane M, marker; Lane 1, *Glu-A3a*; Lane 2, *Glu*-*A3b*; Lane 3, *Glu*-*A3c*; Lane 4, *Glu*-*A3d*; Lane 5, *Glu*-*A3e*; Lane 6, *Glu*-*A3f*; Lane 7, *Glu*-*A3g*; Lane 8, H_2_O. (**E**,**G**): HMW-GS composition Lane 1, Zhoumai 22; Lane 2, *Glu*-*A3a*; Lane 3, *Glu*-*A3b*; Lane 4, *Glu*-*A3c*; Lane 5, *Glu*-*A3d*; Lane 6, *Glu*-*A3e*; Lane 7, *Glu*-*A3f*; Lane 8, *Glu*-*A3g*. (**F**,**H**): Gliadin composition. Lane 1, Zhoumai 23; Lane 2, *Glu*-*A3a*; Lane 3, *Glu*-*A3b*; Lane 4, *Glu*-*A3c*; Lane 5, *Glu*-*A3d*; Lane 6, *Glu*-*A3e*; Lane 7, *Glu*-*A3f*; Lane 8, *Glu*-*A3g*. (**A**,**C**,**E**,**G**) represent NILs under the Zhoumai 22 background; (**B**,**D**,**F**,**H**) represent NILs under the Zhoumai 23 background.

The gluten protein composition of NILs was systematically characterized using molecular marker technology combined with electrophoretic analysis (Figure 1A,B). Using the markers *gluA3a*, *gluA3b*, *gluA3ac*, *gluA3d*, *gluA3e*, *gluA3f*, and *gluA3g*, different DNA fragments were found in seven near-isogenic lines under the genetic background of Zhoumai 22: Lane 1 (529 bp), Lane 3 (894 bp), Lane 5 (573 bp), Lane 9 (967 bp), Lane 11 (158 bp), Lane 13 (552 bp), and Lane 15 (1345 bp), corresponding to *Glu-A3a*, *Glu-A3b*, *Glu-A3a/Glu-A3c*, *Glu-A3d*, *Glu-A3e*, *Glu-A3f*, and *Glu*-*A3g* allelic variants, respectively. To confirm the LMW-GS composition of the NIL A3c, the *gluA3a* molecular marker was employed, yielding no amplification products (Lane 7), thereby validating its allelic variants as *Glu*-*A3c*. NILs in the Zhoumai 23 background exhibited identical LMW-GS compositions at the *Glu*-*A3* locus as those in Zhoumai 22. The *Glu*-*B3* locus was analyzed using *gluB3j* and *gluB3d* molecular markers. In the Zhoumai 22 background, all seven NILs produced a 1500 bp DNA fragment (Figure 1C); the results showed that the LMW-GS composition of the *Glu-B3* locus in this series of materials was *Glu-B3j*. NILs in the Zhoumai 23 background generated a 662 bp fragment (Figure 1D), indicative of the *Glu-B3d* allele. SDS-PAGE and A-PAGE analyses were conducted to assess HMW-GS and gliadin profiles. NILs in the Zhoumai 22 background uniformly exhibited HMW-GS compositions of 1/7 + 8/2 + 12 (Figure 1E) and identical gliadin banding patterns (Figure 1G). Similarly, NILs in the Zhoumai 23 background displayed consistent HMW-GS compositions of 1/7 + 9/4 + 12 (Figure 1F) and gliadin profiles (Figure 1H).

These results demonstrate that NILs within either the Zhoumai 22 or Zhoumai 23 genetic backgrounds differ exclusively in LMW-GS allelic variations at the *Glu-A3* locus while sharing LMW-GSs at *Glu-B3* locus, HMW-GSs, and gliadin compositions. This genetic uniformity establishes these NILs as ideal experimental materials for investigating the functional impacts of *Glu-A3* LMW-GS allelic variations on dough properties and noodle quality. It should be noted that we did not analyze the composition of LMW-GSs at the *Glu-D3* locus because previous studies have shown that the *Glu-D3* locus has a relatively minor impact on quality traits [14,24].

### 3.2. The Effect of LMW-GS Allelic Variations at the Glu-A3 Locus on Protein Content

**Figure 2:**
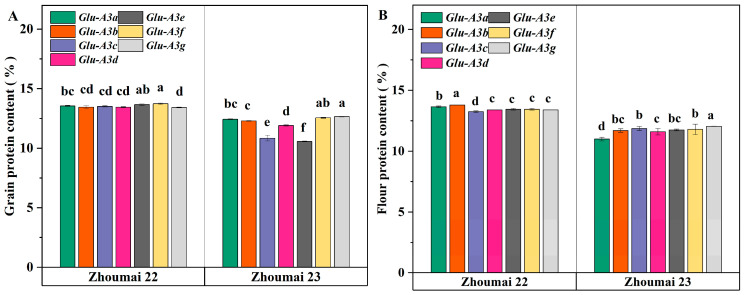
Effect of LMW-GS allelic variations at the *Glu*-*A3* locus on protein content in Zhoumai 22 and Zhoumai 23 backgrounds. (**A**) Grain protein content (%); (**B**) flour protein content (%). Different lowercase letters indicate significant differences at the 0.05 level among NILs at the same genetic background.

The allelic variations of LMW-GSs at the *Glu-A3* locus significantly influence GPC and FPC (Figure 2, *p* < 0.05). Under the Zhoumai 22 background, the rankings of the effect size of each NIL on the GPC (CV = 0.9%) and FPC (CV = 1.3%) were as follows: A3f (13.74%) ≥ A3e (13.66%) ≥ A3a (13.55%) ≥ A3c (13.51%)/A3b (13.45%)/A3d (13.45%) > A3g (13.42%); and A3b (13.80%) > A3a (13.65%) > A3e (13.45%)/A3f (13.45%)/A3d (13.40%)/A3g (13.40%) > A3c (13.25%). In the Zhoumai 23 background, the rankings for the GPC (CV = 7.2%) and FPC (CV = 2.8%) were as follows: A3g (12.65%) ≥ A3f (12.55%) ≥ A3a (12.43%) ≥ A3b (12.29%) > A3d (11.90%) > A3c (10.84%) > A3e (10.56%); and A3g (12.05%) > A3c (11.87%)/A3f (11.8%) > A3e (11.75%)/A3b (11.7%) > A3d (11.6%) > A3a (11.0%). The coefficient of variation (CV) indicates that allelic variations in GPC under the Zhoumai 23 background exhibit greater differences. Notably, the NILs A3c and A3e in Zhoumai 23 showed lower GPC, while their FPC remained relatively high, which may be related to lower flour yields [25]. For ease of comparison, we consider LMW-GS allelic variants ranked in the top two positions for quality traits as better performers, while those in the bottom two are categorized as poorer performers. A3f demonstrates favorable performance in GPC. The relationship between LMW-GSs and protein content remains unclear. Zhang et al. [14], using LMW-GS NILs in the Aroona genetic background, found no significant differences in GPC among *Glu*-*A3* LMW-GS allelic variants (*Glu*-*A3b*, *Glu*-*A3c*, *Glu*-*A3d*, *Glu*-*A3e*, and *Glu*-*A3f*). In contrast, Li et al. [26], utilizing LMW-GS-NILs in the Yanzhan 1 genetic background, observed that the *Glu*-*A3a* allelic variant at the *Glu*-*A3* locus exhibited significantly higher GPC compared to *Glu*-*A3b* and *Glu*-*A3d*. These inconsistencies suggest further studies should be conducted to study the relationship between the protein content and LMW-GS allelic variants.

### 3.3. The Effect of LMW-GS Allelic Variations at the Glu-A3 Locus on Gluten Properties

**Figure 3:**
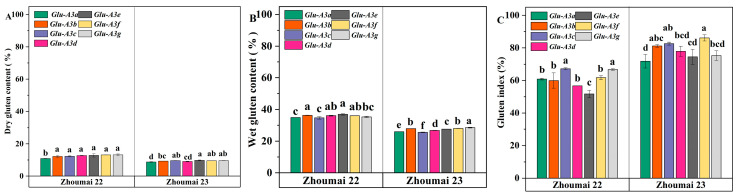
Effect of LMW-GS allelic variations at the *Glu*-*A3* locus on gluten characteristics in Zhoumai 22 and Zhoumai 23 backgrounds. (**A**) dry gluten content (%); (**B**) wet gluten content (%); (**C**) gluten index (%). Different lowercase letters indicate significant differences at the 0.05 level among NILs at the same genetic background.

LMW-GSs connect with HMW-GSs through disulfide bonds to form the gluten network structure, directly influencing gluten quality. The allelic variations of LMW-GSs at the *Glu*-*A3* locus significantly affect gluten characteristics (Figure 3, *p* < 0.05). Under the genetic background of Zhoumai 22, the rankings of the effect size of each NIL on the WGC, DGC, and GI were as follows: A3b (36.34%)/A3e (36.92%) ≥ A3d (36.10%)/A3f (36.04%) ≥ A3g (35.33%) ≥ A3a (34.91%)/A3c (34.70%); A3g (13.19%)/A3f (13.17%)/A3e (12.89%)/A3d (12.77%)/A3c (12.33%)/A3b (12.23%) > A3a (10.90%); and A3c (67.24%)/A3g (66.67%) > A3f (61.63%)/A3a (60.77%)/A3b (59.90%)/A3d (56.68%) > A3e (51.69%). Under the genetic background of Zhoumai 23, the rankings of the effect size of each NIL on the WGC, DGC, and GI were as follows: A3g (28.55%) > A3f (28.04%)/A3b (27.89%) > A3e (27.57%) > A3d (26.76%) > A3a (25.93%) > A3c (25.58%); A3e (9.80%) ≥ A3c (9.64%)/A3g (9.57%)/A3f (9.51%) ≥ A3b (9.32%) ≥ A3d (9.09%) ≥ A3a (8.78%); and A3f (86.09%) ≥ A3c (82.62%)/A3b (81.21%) ≥ A3d (77.89%)/A3g (75.28%) ≥ A3e (74.48%) ≥ A3a (71.83%).

Evidently, under both genetic backgrounds, A3a and A3c exhibited poorer performance in WGC, while A3g showed better DGC and A3a performed poorly in DGC. For GI, A3c demonstrated favorable results, whereas A3e performed poorly. Zhou et al. [16], using NILs in the Xiaoyan 22 genetic background, found no significant differences in gluten content among *Glu*-*A3* LMW-GS allelic variants (*Glu*-*A3a*, *Glu*-*A3b*, *Glu*-*A3c*, and *Glu*-*A3e*), with the ranking of GI effects being *Glu*-*A3b* > *Glu*-*A3c* > *Glu*-*A3a*/*Glu*-*A3e*. *Glu*-*A3e* consistently exerted a negative impact on GI across three genetic backgrounds (Xiaoyan 22, Zhoumai 22, and Zhoumai 23), indicating that its detrimental effect is likely independent of genetic background. However, in the case of gluten content, the previous results showed inconsistency with our results, which may be due to different genetic backgrounds [15,16].

### 3.4. The Effect of LMW-GS Allelic Variations at the Glu-A3 Locus on Dough Farinograph Properties

Wheat LMW-GSs significantly affect dough properties. As shown in Table 2, under the Zhoumai 22 background, the rankings of the effect size of each NIL on the water absorption (WA), development time (DT), stability time (ST), degree of softening (DS), and farinograph quality number (FQN) were as follows: A3a > A3e > A3b > A3f > A3g > A3c/A3d; A3b > A3g > A3a > A3d/A3e/A3f > A3c; A3d > A3g > A3b > A3a > A3c/A3f > A3e; A3c > A3e > A3f > A3a > A3d > A3g > A3b; and A3d > A3b > A3g > A3a > A3f > A3e > A3c, respectively. Under the Zhoumai 23 background, the rankings were as follows: A3e > A3g > A3d/A3f > A3b > A3c > A3a (WA); A3b > A3f > A3c/A3g > A3e > A3d > A3a (DT); A3b > A3f/A3c/A3g > A3d > A3a > A3e (ST); A3e > A3d > A3a > A3c > A3b > A3f > A3g (DS); and A3b > A3c/A3g > A3f > A3d > A3e > A3a (FQN). Under the two genetic backgrounds, A3e demonstrated better performance in WA, while A3c performed poorly; A3b showed longer DT; A3e exhibited a favorable DS, whereas A3g performed poorly in DS; and A3b excelled in FQN, while A3e underperformed. Based on their physical meanings, these parameters are categorized into water absorption characteristics (WA) and mixing tolerance characteristics (DT, ST, DS, and FQN). Within the same genetic background, NILs exhibited low variation in WA (CV < 3%), while mixing tolerance parameters—ST (CV = 16.58%–21.36%), DT (CV = 9.24%–14.47%), and FQN (CV = 10.53%–11.69%)—showed higher variability, indicating that *Glu*-*A3* LMW-GS allelic variations minimally affect WA but predominantly influence mixing tolerance. Zhou et al. [16] observed similarly low WA variation (CV = 0.5%) among *Glu*-*A3b*, *Glu*-*A3a*, *Glu*-*A3c*, and *Glu*-*A3e*. Jin et al. [15], using Aroona-background NILs, ranked the impact of *Glu*-*A3* alleles on optimal dough mixing time as *Glu*-*A3b* > *Glu*-*A3d* > *Glu*-*A3f* > *Glu*-*A3c* > *Glu*-*A3e*. Zhou et al. [16] also demonstrated that *Glu*-*A3b* in Xiaoyan 22-background NILs outperformed *Glu*-A3a, *Glu*-*A3c*, and *Glu*-*A3e* in mixing tolerance. Our results further proved the superiority of *Glu-A3b* for the mixing tolerance. Across four genetic backgrounds (Aroona, Xiaoyan 22, Zhoumai 22, and Zhoumai 23), *Glu*-*A3b* consistently exhibited superior mixing tolerance, establishing it as a superior subunit for improving dough processing properties.

### 3.5. The Effect of LMW-GS Allelic Variations at the Glu-A3 Locus on FWN Quality Characteristics

#### 3.5.1. Cooking Characteristics of FWNs

The cooking characteristics mainly include the optimal cooking time (OCT), water absorption ratio (WAR), and cooking loss ratio (CLR). As shown in Table 3, the LMW-GS allelic variations at the *Glu*-*A3* locus differentially influenced three parameters across the two genetic backgrounds. Under the Zhoumai 22 background, no significant differences were observed among NILs in OCT and CLR, while their impact on WAR was ranked as A3f ≥ A3a/A3b/A3c/A3g ≥ A3d/A3e. In the Zhoumai 23 background, WAR showed no significant variation, while the rankings for OCT and CLR were as follows: A3a/A3b/A3c/A3d/A3e/A3g ≥ A3f and A3a > A3c/A3d/A3e/A3f/A3g > A3b, respectively. The OCT of A3f was significantly lower than the other subunits. It is worth noting that A3f showed a lower OCT, and we do not know the specific reason for this difference, which may be affected by the genetic background [27].

#### 3.5.2. Textural Characteristics of Fresh Wet Noodles (FWNs)

From Table 4, it can be seen that the allelic variation of LMW-GSs at the *Glu-A3* locus has a significant effect on hardness and chewiness in both genetic backgrounds, while cohesiveness and resilience only have a significant effect in the Zhoumai 23 background, and the two genetic backgrounds have no significant effect on adhesiveness and springiness. This may be related to the genetic background differences between the two varieties. In both genetic backgrounds, A3b showed greater hardness. A3g/A3f of Zhoumai 23 and A3b/A3a of Zhoumai 22 showed greater chewiness. For noodle products, moderate hardness is considered to have the best quality. However, moderate hardness does not give a specific hardness range. In this paper, different allelic variations mainly affect the hardness and chewiness. These results can provide theoretical guidance for the improvement of noodle hardness and chewiness. Jin et al. [15], using NILs in the Aroona genetic background and sensory evaluation, reported no significant differences in cooked noodle hardness or viscoelasticity among *Glu*-*A3* LMW-GS allelic variants (*Glu*-*A3b*, *Glu*-*A3c*, *Glu*-*A3d*, *Glu*-*A3e*, and *Glu*-*A3f*). However, Zhou et al. [16], employing texture analyzer-based assessments on Xiaoyan 22-derived NILs, observed that *Glu*-*A3b* exhibited lower noodle hardness compared to *Glu*-*A3a* but outperformed *Glu*-*A3c* and *Glu*-*A3e*, while *Glu*-*A3c* and *Glu*-*A3e* showed better adhesiveness, cohesiveness, and springiness relative to *Glu*-*A3a* and *Glu*-A3b. The inconsistency with previous findings suggests that the effect of LMW-GS allelic variants at the *Glu-A3* locus on noodle texture is strongly influenced by the genetic background, and may also be related to differences in experimental methods, such as the difference in the formula, whether salt is added or not, the difference in the size of noodles, and the difference in the quality evaluation (sensory evaluation or texture analyzer) [15,16].

### 3.6. The Effect of LMW-GS Allelic Variations at the Glu-A3 Locus on Textural Characteristics of Frozen Cooked Noodles (FZNs)

The influence of LMW-GSs on the textural properties of FZNs remains unexplored. Our investigation revealed that allelic variations at the *Glu*-*A3* locus significantly affected the texture of FZNs (Table 5). The LMW-GS allelic variations at the *Glu-A3* locus had significant effects on the hardness and chewiness of frozen cooked noodles in both genetic backgrounds but had no significant effect on cohesiveness and resilience. It had a significant effect on adhesiveness and springiness under the background of Zhoumai 23, which may be related to the genetic background differences between the two varieties. It is worth noting that A3g showed greater hardness and chewiness in both backgrounds. FZNs made of NILs containing *Glu-A3g* can maintain good processing quality in a low temperature environment and reduce the quality decline of noodles caused by freezing. This is of great significance in the processing and storage of frozen food, which can prolong the shelf life of products, reduce loss, and improve economic benefits. Maintaining good hardness and elasticity after the rehydration of frozen cooked noodles improves the taste and quality, and increases consumer satisfaction. Tao et al. [28] demonstrated that, compared to HMW-GSs, the complexes formed by LMW-GSs with wheat starch granule surface proteins exhibit higher cryoprotective capacity, which is critical for maintaining frozen dough quality. This study further clarified the influence patterns of LMW-GS allelic variations on the quality of FZNs from the perspective of these variations.

## 4. Conclusions

In order to clarify the influence of different LWW-GS allelic variations at the *Glu-A3* locus on fresh wet noodles and frozen cooked noodles, two groups of NILs from Zhoumai 22 and Zhoumai 23 were obtained and used in this research. *Glu-A3f* was selected to improve grain protein content, *Glu-A3g* was selected to improve dry gluten content and the hardness and chewiness of frozen cooked noodles, and *Glu-A3b* was selected to prolong dough mixing resistance and increase fresh noodle hardness, while *Glu-A3e*, with a negative effect on the gluten index, should be avoided. These results can provide LWW-GS options for the improvement of wheat varieties and flour specifically for noodles.

## Figures and Tables

**Table 1:** The information of STS markers used to distinguish the allele variations *Glu*-*A3a*, *Glu-A3b*, *Glu-A3c*, *Glu-A3e*, *Glu-A3f*, *Glu-A3g*, *Glu*-*B3d*, and *Glu-B3j*.

Marker	Sequence of Primers (5′→3′)	Fragment Size (bp)	Annealing Temperature (°C)	Reference
*gluA3a*	F: AAACAGAATTATTAAAGCCGG	529	55	Wang et al. [12,20]
	R: GGTTGTTGTTGTTGCAGCA			
*gluA3b*	F: TTCAGATGCAGCCAAACAA	894	57	
	R: GCTGTGCTTGGATGATACTCTA			
*gluA3ac*	F: AAACAGAATTATTAAAGCCGG	573	58	
	R: GTGGCTGTTGTGAAAACGA			
*gluA3d*	F: TTCAGATGCAGCCAAACAA	967	56	
	R: TGGGGTTGGGAGACACATA			
*gluA3e*	F: AAACAGAATTATTAAAGCCGG	158	57	
	R: GGCACAGACGAGGAAGGTT			
*gluA3f*	F: AAACAGAATTATTAAAGCCGG	552	57	
	R: GCTGCTGCTGCTGTGTAAA			
*gluA3g*	F: AAACAGAATTATTAAAGCCGG	1345	57	
	R: AAACAACGGTGATCCAACTAA			
*gluB3d*	F: CACCATGAAGACCTTCCTCA	662	58	
	R: GTTGTTGCAGTAGAACTGGA			
*gluB3j*	F: GGAGACATCATGAAACATTTG	1500	58	Francis et al. [19]
	R: CTGTTGTTGGGCAGAAAG			

**Table 2:** Effect of LMW-GS allelic variations at the *Glu*-*A3* locus on dough farinograph properties in Zhoumai 22 and Zhoumai 23 backgrounds.

Background	NIL	Water Absorption/%	Development Time/min	Stability Time/min	Degree of Softening/BU	Farinograph Quality Number/mm
Zhoumai 22	A3a	63.2	2.5	1.6	158	31
	A3b	62.2	2.9	1.7	141	36
	A3c	59.6	2.2	1.3	184	27
	A3d	59.6	2.4	2.1	155	37
	A3e	62.7	2.4	1.2	170	29
	A3f	61.8	2.4	1.3	167	30
	A3g	61.3	2.7	1.9	152	34
CV(%)		2.32	9.24	21.36	8.68	11.69
Zhoumai 23	A3a	56.9	1.7	1.7	163	27
	A3b	59.9	2.5	2.4	153	35
	A3c	59.8	2.3	2.2	154	34
	A3d	60.2	1.8	1.8	164	29
	A3e	61.6	2	1.5	197	28
	A3f	60.2	2.4	2.2	152	33
	A3g	61.2	2.3	2.2	149	34
CV(%)		2.52	14.47	16.58	10.23	10.53

**Table 3:** Effect of LMW-GS allelic variations at the *Glu*-*A3* locus on cooking characteristics of FWNs in Zhoumai 22 and Zhoumai 23 backgrounds.

Background	NIL	Optimal Cooking Time (s)	Water Absorption Ratio (%)	Cooking Loss Ratio (%)
Zhoumai 22	A3a	202.50 ± 10.61 a	116.78 ± 6.58 ab	8.97 ± 0.27 a
	A3b	195.00 ± 0.00 a	112.67 ± 0.22 ab	9.76 ± 0.61 a
	A3c	195.00 ± 0.00 a	113.22 ± 1.45 ab	10.19 ± 0.47 a
	A3d	195.00 ± 0.00 a	107.09 ± 0.96 b	9.62 ± 1.59 a
	A3e	187.50 ± 10.61 a	111.54 ± 6.45 b	8.48 ± 0.10 a
	A3f	195.00 ± 0.00 a	122.05 ± 5.39 a	9.68 ± 1.58 a
	A3g	202.50 ± 10.61 a	113.56 ± 1.31 ab	9.39 ± 0.19 a
Zhoumai 23	A3a	202.50 ± 10.61 a	114.40 ± 1.82 a	10.92 ± 1.08 a
	A3b	195.00 ± 0.00 a	117.23 ± 4.52 a	9.37 ± 0.34 b
	A3c	195.00 ± 0.00 a	113.85 ± 3.58 a	10.38 ± 0.91 ab
	A3d	195.00 ± 0.00 a	111.68 ± 4.17 a	10.19 ± 0.12 ab
	A3e	195.00 ± 0.00 a	112.40 ± 2.87 a	10.16 ± 0.04 ab
	A3f	180.00 ± 0.00 b	114.09 ± 9.05 a	10.76 ± 0.29 ab
	A3g	195.00 ± 0.00 a	110.98 ± 2.49 a	10.24 ± 0.44 ab

Different lowercase letters indicate significant differences at the 0.05 level among NILs at the same genetic background.

**Table 4:** Effect of LMW-GS allelic variations at the *Glu*-*A3* locus on textural characteristics of cooked FWNs in Zhoumai 22 and Zhoumai 23 backgrounds.

Background	NIL	Hardness (g)	Adhesiveness (g.s)	Springiness (%)	Cohesiveness (%)	Resilience (%)	Chewiness (g)
Zhoumai 22	A3a	281.15 ± 0.87 b	−1.09 ± 0.32 a	85.53 ± 0.50 a	65.71 ± 0.92 a	37.60 ± 1.10 a	157.95 ± 0.80 a
	A3b	299.49 ± 2.01 a	−2.25 ± 0.24 a	85.77 ± 1.81 a	63.30 ± 3.70 a	35.21 ± 2.63 a	162.79 ± 13.89 a
	A3c	300.80 ± 2.75 a	−2.44 ± 0.79 a	84.16 ± 2.05 a	59.69 ± 0.83 a	32.57 ± 0.79 a	151.42 ± 7.49 ab
	A3d	277.81 ± 0.76 bc	−1.38 ± 0.18 a	84.90 ± 0.19 a	63.64 ± 0.93 a	35.06 ± 1.00 a	150.08 ± 2.06 ab
	A3e	270.87 ± 7.42 c	−1.72 ± 0.74 a	82.85 ± 0.97 a	58.70 ± 2.00 a	32.02 ± 2.72 a	132.03 ± 9.99 b
	A3f	252.05 ± 1.52 d	−1.46 ± 1.03 a	83.51 ± 1.70 a	60.37 ± 5.97 a	34.07 ± 4.66 a	127.09 ± 14.47 b
	A3g	279.83 ± 4.32 b	−1.42 ± 0.43 a	84.04 ± 1.03 a	62.59 ± 4.20 a	35.19 ± 3.68 a	147.10 ± 9.34 ab
Zhoumai 23	A3a	264.80 ± 1.93 e	−3.47 ± 1.20 a	84.37 ± 2.88 a	57.51 ± 6.15 b	31.76 ± 5.89 b	128.49 ± 10.44 c
	A3b	316.63 ± 0.48 a	−1.88 ± 0.30 a	84.77 ± 1.24 a	62.58 ± 0.19 ab	35.12 ± 0.43 ab	167.91 ± 2.26 ab
	A3c	297.98 ± 0.40 cd	−2.21 ± 0.12 a	85.83 ± 0.07 a	64.04 ± 0.03 ab	37.12 ± 0.27 ab	163.72 ± 0.41 ab
	A3d	311.83 ± 6.07 ab	−2.16 ± 0.59 a	84.98 ± 0.66 a	62.18 ± 2.42 ab	35.75 ± 3.30 ab	164.76 ± 10.96 ab
	A3e	291.51 ± 6.75 d	−2.63 ± 1.90 a	84.95 ± 0.12 a	61.60 ± 3.99 ab	35.29 ± 4.87 ab	152.52 ± 13.22 b
	A3f	301.94 ± 10.38 bcd	−1.34 ± 0.32 a	85.60 ± 0.18 a	66.04 ± 0.62 a	39.91 ± 0.72 a	170.63 ± 4.65 ab
	A3g	307.95 ± 1.52 abc	−1.35 ± 0.55 a	85.19 ± 0.12 a	66.43 ± 0.26 a	39.57 ± 0.85 ab	174.26 ± 0.33 a

Different lowercase letters indicate significant differences at the 0.05 level among NILs with the same genetic background.

**Table 5:** Effects of LMW-GS allelic variations at the *Glu*-*A3* locus on the texture quality of FZNs under the backgrounds of Zhoumai 22 and Zhoumai 23.

Background	NIL	Hardness (g)	Adhesiveness (g.s)	Springiness (%)	Cohesiveness (%)	Resilience (%)	Chewiness (g)
Zhoumai 22	A3a	252.91 ± 1.31 b	−0.34 ± 0.03 a	82.50 ± 0.40 a	47.97 ± 0.76 a	32.01 ± 0.14 a	100.12 ± 1.73 ab
	A3b	235.68 ± 4.13 bc	−0.85 ± 0.51 a	82.95 ± 1.28 a	48.74 ± 7.98 a	31.54 ± 2.15 a	95.51 ± 18.75 ab
	A3c	247.38 ± 0.15 b	−0.76 ± 0.61 a	83.87 ± 0.30 a	48.89 ± 8.11 a	33.17 ± 1.76 a	101.43 ± 17.38 ab
	A3d	197.97 ± 0.00 d	−0.86 ± 0.87 a	81.96 ± 0.22 a	43.27 ± 0.99 a	34.87 ± 6.96 a	70.11 ± 1.76 b
	A3e	213.21 ± 14.96 cd	−1.43 ± 0.50 a	81.45 ± 1.04 a	39.15 ± 0.60 a	28.41 ± 4.29 a	67.83 ± 4.51 b
	A3f	200.55 ± 20.81 d	−0.85 ± 0.72 a	80.75 ± 1.07 a	48.44 ± 1.87 a	30.19 ± 1.24 a	78.25 ± 6.10 ab
	A3g	287.89 ± 5.67 a	−0.59 ± 0.49 a	83.58 ± 2.82 a	50.53 ± 15.19 a	32.71 ± 7.14 a	122.01 ± 37.96 a
Zhoumai 23	A3a	197.98 ± 16.69 d	−1.29 ± 0.99 b	80.35 ± 1.15 b	46.54 ± 4.76 a	30.32 ± 4.28 a	75.03 ± 14.32 b
	A3b	273.27 ± 1.53 b	−0.86 ± 0.27 ab	84.80 ± 1.39 a	53.05 ± 7.07 a	31.39 ± 0.41 a	123.19 ± 17.51 a
	A3c	262.32 ± 0.48 bc	−0.87 ± 0.50 ab	82.67 ± 1.54 ab	51.51 ± 1.75 a	29.68 ± 3.09 a	111.76 ± 5.72 ab
	A3d	258.55 ± 6.99 bc	−1.37 ± 0.10 b	83.20 ± 0.23 ab	49.80 ± 2.49 a	25.61 ± 3.03 a	107.19 ± 8.67 ab
	A3e	290.92 ± 6.10 a	−0.45 ± 0.22 a	82.57 ± 2.55 ab	45.75 ± 14.51 a	31.63 ± 5.71 a	110.21 ± 35.62 ab
	A3f	254.07 ± 0.68 c	−1.13 ± 1.14 ab	82.61 ± 0.89 ab	50.99 ± 0.17 a	28.22 ± 0.25 a	107.15 ± 0.56 ab
	A3g	295.96 ± 2.27 a	−0.57 ± 0.11 ab	83.97 ± 0.67 a	53.76 ± 6.26 a	33.32 ± 3.00 a	133.94 ± 15.10 a

Different lowercase letters indicate significant differences at the 0.05 level among NILs at the same genetic background.

## Data Availability

The original contributions presented in the study are included in the article/Supplementary Materials. Further inquiries can be directed to the corresponding authors.

## Supplementary Materials

The following supporting information can be downloaded at: https://www.mdpi.com/article/10.3390/foods14091546/s1, Table S1: Recurrent and non-recurrent parents used for 2 sets of NILs cultivation.
