# Supplementary material for: Effects of LMW-GS Allelic Variations at the Glu-A3 Locus on Fresh Wet Noodle and Frozen Cooked Noodle Quality
Source: Foods. 2025 Apr 28;14(9):1546. doi: 10.3390/foods14091546 (PMC12071937; doi:10.3390/foods14091546)
Supplement: Supplementary file 1 [file foods-14-01546-s001.zip › foods-3573087-supplementary.pdf]

Table S1 Recurrent and non-recurrent parents used for 2 sets of NILs cultivation

**Recurrent parents**	**NIL**	**Non-recurrent parents**	***Glu-A3* locus LMW-GS**	***Glu-B3* locus LMW-GS**
Zhoumai 22 (*Glu-A3d*, *Glu-B3j*)	A3a	Chinese Spring (*Glu-A3a*, *Glu-B3a*)	*a*	*j*
	A3b	Shandong 413863 (*Glu-A3b*, *Glu-B3j*)	*b*	*j*
	A3c	Yuwai 69 (*Glu-A3c, Glu-B3d*)	*c*	*j*
	A3d	CA9641 (*Glu-A3d, Glu-B3h*)	*d*	*j*
	A3e	Jinnong 207 (*Glu-A3e, Glu-B3j*)	*e*	*j*
	A3f	Yuandong 6 (*Glu-A3f, Glu-B3j*)	*f*	*j*
	A3g	Gaocheng 8901 (*Glu-A3g, Glu-B3i*)	*g*	*j*
Zhoumai 23 (*Glu-A3d*, *Glu-B3d*)	A3a	Chinese Spring (*Glu-A3a, Glu-B3a*)	*a*	*d*
	A3b	Shandong 413863 (*Glu-A3b, Glu-B3j*)	*b*	*d*
	A3c	CA9719 (*Glu-A3c, Glu-B3h*)	*c*	*d*
	A3d	Nongda 116 (*Glu-A3d, Glu-B3d*)	*d*	*d*
	A3e	Jinnong 207 (*Glu-A3e, Glu-B3j*)	*e*	*d*
	A3f	Nongda 3213 (*Glu-A3f, Glu-B3j*)	*f*	*d*
	A3g	Gaocheng 8901 (*Glu-A3g, Glu-B3i*)	*g*	*d*
